# Evaluation of a Major Surface Antigen of *Babesia microti* Merozoites as a Vaccine Candidate against Babesia Infection

**DOI:** 10.3389/fmicb.2017.02545

**Published:** 2017-12-19

**Authors:** Suqin Man, Yongfeng Fu, Yue Guan, Meng Feng, Ke Qiao, Xueping Li, Hongjian Gao, Xunjia Cheng

**Affiliations:** ^1^Department of Medical Microbiology and Parasitology, School of Basic Medical Sciences, Fudan University, Shanghai, China; ^2^Department of Electron Microscopy, School of Basic Medical Science, Fudan University, Shanghai, China; ^3^Institute of Biomedical Sciences, Fudan University, Shanghai, China

**Keywords:** babesiosis, *Babesia microti*, surface antigen, vaccine, cytokines

## Abstract

*Babesia* species are tick-borne intraerythrocytic protozoa that cause babesiosis in humans worldwide. No vaccine has yet proven effective against *Babesia* infection. Surface antigens of merozoites are involved in the invasion of erythrocytes by *Babesia*. Surface antigens may be presented by both babesial sporozoites and merozoites and provide a general target for antibody-mediated inhibition of erythrocyte invasion. Here we evaluated a major surface antigen of *B. microti* merozoites, BMSA, as a potential vaccine to prevent babesiosis. Our data indicated that *bmsa* is transcribed during different phases, including ring form, amoeboid form, and merozoites, and that its expression is significantly increased in mature merozoites. The protein was found to be located in the membrane of *B. microti* and in the cytoplasm of infected erythrocytes. The immune response induced by BMSA had a significant inhibitory effect on parasite invasion of the host erythrocytes (83.3% inhibition of invasion) and parasite growth *in vivo*. The levels of parasitemia significantly decreased after BMSA vaccination when mice were infected with babesia parasite. Importantly, protective immunity was significantly related to the upregulation of the Th17 cytokine interleukin-17, the Th1 cytokine interleukin-12p70 and the Th2 cytokines, such as interleukin-4, -6, and -10. Ingenuity Pathway Analysis indicated that interleukin-17 facilitated the secretion of Th2 cytokines, such as interleukin-10, -4, and -6, thereby inducing a predominately Th2 protective immune response and promoting the expression a high level of special IgG1 against *Babesia* infection. Further, an anti-BMSA monoclonal antibody successfully protected NOD/SCID mice from a challenge with *B. microti*. Taken together, our results indicated that BMSA induces a protective immune response against *Babesia* infection and may serve as a potential vaccine.

## Introduction

*Babesia microti* is a tick-borne intraerythrocytic protozoan parasite belonging to the phylum *Apicomplexa*. *B. microti* causes babesiosis in animals and humans worldwide (Vannier and Krause, 2012). Babesiosis predominantly affects animals, although occasional cases of babesiosis in humans have attracted increasing attention. In immunocompetent persons, babesiosis is rarely detected as patients are usually asymptomatic or present with mild symptoms that are often self-resolving. Nevertheless, babesiosis can be life-threatening in certain populations, such as neonates/infants or immunocompromised patients (Gabrielli et al., 2016).

The parasites have a sexual stage in ticks and an asexual intraerythrocytic cycle in mammalian erythrocytes (Gray et al., 2010; Vannier and Krause, 2012). Parasites that live in the erythrocytes have rather ingenious ways of gaining entry into these cells; thus, escaping the host immune system. The blood stage of this parasite causes the pathobiology called babesiosis by invading and subsequently modifying human erythrocytes.

During parasite invasion and development within host cells, the parasite produces surface proteins that allow it to adhere to and invade erythrocytes where it survives, grows, and develops. Surface proteins generally play a critical role in facilitating parasite invasion, host cell remodeling, nutrient acquisition, waste disposal, environmental sensing, and protection from the innate defense mechanisms. In the host, these proteins are targeted by the humoral immune response or they activate a T-effector cell response (Hines et al., 1995; Suarez et al., 2000). The surface proteins present in early transcribed membrane proteins of *B. microti* include the merozoite surface antigens, the variable merozoite surface antigen family, and the rhoptry-associated proteins (Hines et al., 1995; Suarez et al., 2000; Mosqueda et al., 2002a,b; Jaramillo Ortiz et al., 2016). Such surface antigens may be useful for developing a diagnostic test for babesiosis as well as a vaccine.

Vaccine was regarded as a prospective strategy against babesia infection. Several proteins had yet been evaluated, including heat shock protein-70 (BmHSP-70), apical membrane protein 1 (BmAMA1), profiling (PROF), methionine aminopeptidase 1 (BmMetAP1) and rhoptry neck protein 2 (BmRON2), all of them were analyzed as vaccines in recent years (Terkawi et al., 2009; Moitra et al., 2015; Munkhjargal et al., 2016a,b; Wang et al., 2017). But vaccination with BmAMA1and BmRON2 exhibited a limited protection against *B. microti* challenge. Immunization with BmMetAP1, BmHSP-70 or PROF elicited the modest protection from the infection of *B. microti*. Thus a novel target served as vaccine candidate worth exploring.

A previous study screened a genomic expression library with patient serum pools and identified 17 *B. microti* antigens that are targets of humoral immune responses in humans with babesiosis (Lodes et al., 2000). A screen of a *B. microti* cDNA expression library using sera from immunized hamsters identified a novel 33-kDa secreted antigen of *B. microti* (BmSA1) (Luo et al., 2011). Recently, Priest *et al*., used classical antigen identification techniques to demonstrate that the BMN1-9/BmSA1 antigen is membrane-associated and is commonly detected in the sera of humans infected with *B. microti* (Priest et al., 2012). However, the antigenicity, immunogenicity, function, and subcellular localization of these surface antigens are not clearly understood. Compiling this information will help to elucidate invasion on a molecular level and to fully understand the mechanism by which *Babesia* invades a host cell. Recombinant surface antigens should induce antibody production in animal models or provide protection from a parasite challenge.

The current study determined the subcellular localization of a surface antigen of *B. microti*, and we hypothesized that this antigen may serve as a potential recombinant vaccine that will improve the extent of humoral immune responses. Further, this study analyzed the significance of humoral responses and cell-mediated immune responses in protecting against *Babesia* infection.

## Materials and methods

### Ethics statement

All animal experiments were carried out in strict accordance with the Animal Welfare Act and the guidelines of the Regulations for the Administration of Affairs Concerning Experimental Animals (1988.11.1). All procedures were approved by the Institutional Animal Care and Use Committee of Fudan University, China (Permit Number: 201202019). All efforts were made to minimize suffering.

### Animals and *Babesia*

BALB/c and NOD/SCID female mice, 6–8 weeks of age, were purchased from SLAC Laboratory Animal (Shanghai, China). The Peabody mjr strain of *B. microti* was obtained from ATCC and maintained through infection of BALB/c by intraperitoneal infection of parasite-erythrocytes. Briefly, mice were intraperitoneally administered 1 × 10^7^ erythrocytes infected with *B. microti*.

Parasitemia was detected in thin blood smears stained with Giemsa using a light microscope. Parasitemia was further evaluated using flow cytometry with SYBR Green I dye (Somsak et al., 2012). Briefly, 2 μl of blood obtained from the tail of mice infected with *B. microti* was collected into a 1.5 ml tube containing 500 μl of a premixed stock of glucose-PBS-EDTA (pH 7.4). SYBR Green I was added to the tube at a 6 × concentration and the mixture was incubated at 37°C in the dark for 30 min. Parasitemia was determined using a FACSCalibur (BD).

### Preparation of recombinant BMSA (rBMSA)

Total RNA from *B. microti* was purified from the peripheral blood cells of infected BALB/c mice using an RNeasy Plus Mini Kit (Qiagen). One microgram of purified total RNA was synthesized to cDNA using a Primescript 1st strand cDNA synthesis kit (Takara). The BMSA fragment was amplified using PCR with the sense primer 5′-GGT CAT TCA AAC CAA CCA TAA TCA C-3′ and the antisense primer 5′-GGT TA GA ATA GAA ACA TAG CGA CCG-3′. The PCR was performed as follows: denaturation at 94°C for 3 min, denaturation at 94°C for 30 s, annealing at 55°C for 30 s, polymerization at 68°C for 90 s, and extension for 7 min. The target plasmids were used to transform *Escherichia coli* BL21 Star (DE3) pLysS, and the recombinant protein was purified by a Ni-NTA His•Bind Resin kit (Novagen) following the manufacturer's instructions. Fifty micrograms of the recombinant BMSA were intraperitoneally injected into BALB/c mice to test for endotoxicity.

### Western blot analysis

To produce monoclonal antibodies against BMSA, 50 μg of rBMSA (1 mg/ml) was mixed with 50 μl of complete Freund's adjuvant (the first time) or incomplete Freund's adjuvant (subsequent boost interval after 14 days) and injected into the peritoneal cavity of 6-week-old BALB/c mice. Mice splenocytes were fused with X63Ag8.653 myeloma cells (a gift from Dr. Hiroshi Tachibana, Tokai University) using PEG1500. Hybridoma cells were screened using hypoxanthine-aminopterin-thymidine and hypoxanthine-thymidine selective medium before intraperitoneal injection into BALB/c mice to prepare ascites containing monoclonal antibodies against BMSA. The ascitic fluid containing monoclonal antibodies was purified using an Affi-gel protein A MAPS II kit (Bio-Rad) in accordance with the manufacturer's instructions. The subtype of the monoclonal antibody was identified using a Mouse Typer Sub-Isotyping Kit (Bio-Rad) according to the manufacturer's instructions.

Western blotting was performed as previously described (Potala and Verma, 2010). The purified recombinant BMSA protein and crude *B. microti* antigen were separated using SDS-PAGE and proteins were transferred from the gel to a polyvinylidene difluoride membrane (PVDF) for immunoblotting. After blocking with 3% skim milk, the membrane was incubated with serum from BALB/c mice infected with *B. microti* or treated with the monoclonal antibody. After each incubation, the membrane was washed with PBST and finally developing by an enhanced HRP-DAB substrate detection kit (Tiangen Biotech).

### Subcellular localization of BMSA

Anticoagulated blood (sodium citrate) collected from mice infected with *B. microti* with approximately 30% parasitemia was fixed with 4% PFA-PBS and smeared on slides using cytospin centrifugation (Thermo Fisher Scientific). Indirect immunofluorescence staining was performed with primary antibody of anti-BMSA monoclonal antibody and then Alexa Fluor 488-conjugated goat anti-mouse IgG as the secondary antibody. Propidium iodide was used for counterstaining, and the slides were examined using a confocal microscope (Man et al., 2016).

An immunoelectron microscopy assay was performed to observe the localization of BMSA. For this purpose, erythrocytes infected with *B. microti* were fixed with paraformaldehyde and glutaraldehyde in PBS at 4°C for 2 h, 1% osmium tetroxide at 4°C for 2 h, and embedded in 618 epoxy resin at 37°C for 2 h, at 45°C for 3 h, and at 60°C for 24 h after dehydration. Samples were incubated with the anti-BMSA monoclonal antibody (1:100) and incubated with the goat anti-mouse IgG conjugated to 5 nm gold particles at room temperature for 2 h. The samples were observed using a transmission electron microscope [JEOL JEM-1230 (80 KV)].

### Quantification of BMSA expression using real-time PCR

Infected erythrocytes with 30–40% parasitemia were collected, anticoagulated with sodium citrate, and washed with medium. The sample was treated with 5% sorbitol and washed with medium three times. The hematocrit was adjusted to 5%, and the erythrocyte suspension was added to a 24-well plate. Erythrocytes were cultured at 37°C with 5% CO_2_, 5% O_2_, and 90% N_2_ and sampled every 4 h. Erythrocyte smears were prepared by collecting erythrocytes at different times.

Total RNA was extracted from erythrocytes infected with *B. microti* after synchronous culture for 0, 4, 8, 12, 16, 20, 24, 28, 32, and 36 h. Real-time PCR was performed in triplicate using primers specific for BMSA (forward primer 5′- AAA GAA TTT GAT GAA CGC AAG-3′ and reverse primer 5′- TTA ACT TCA CCG ACT GCA T-3′) and for GAPDH (forward primer 5′-CAT GTG GAT GTT GTT GCA AT-3′ and reverse primer 5′- AGC TTA ACA GTC TTG CCA T−3′) using SYBR *Premix Ex TaqTM* (Perfect Real Time) (Takara).

### Immunization with recombinant BMSA

Sixteen BALB/c mice from each group were vaccinated with purified recombinant BMSA antigen and complete Freund's adjuvant, and boosted two times with recombinant BMSA antigen and incomplete Freund's adjuvant. The adjuvant control group was vaccinated with Tris-HCl and adjuvant. The Tris-HCl control group of animals was intraperitoneally injected with Tris-HCl only. Two weeks after final immunization, following the detection of the antibody titer in the serum, mice were intraperitoneally injected with 1 × 10^7^ erythrocytes infected with *B. microti*. Parasitemia was monitored every 2 days using flow cytometry. Splenocytes were obtained from four infected mice on days 0, 7, 14, and 21 and were stimulated *in vitro* with PBS, BMSA, or concanavalin (ConA) to detect cytokines. Passive immunization assays were performed using anti-*B. microti* monoclonal antibodies. Two groups of NOD/SCID mice were intraperitoneally administered a total of 5 mg of monoclonal antibody in PBS on day −1, 0, and +1. On day 0, mice were challenged with 1 × 10^5^ erythrocytes infected with *B. microti*. The presence of parasitemia was evaluated every 2 days using flow cytometry and Giemsa-stained smears.

### Detection of cytokines and antibodies in serum and medium

After three rounds of immunization with the recombinant protein, mice were challenge with 1 × 10^7^ erythrocytes infected with *B. microti*. Splenocytes obtained from every group on days 0, 7, 14, and 21 were cultured *in vitro* for 72 h and the culture supernatant was collected. Blood was harvested from each immunized and control mouse on days 0, 7, 14, and 21 after challenge. Serum was obtained by centrifugation for 30 min at 3,000 g. The levels of individual cytokines, chemokines, colony-stimulating factor, and antibodies were determined using a Mouse Cytokine/Chemokine or Immunoglobulin Isotyping Magnetic Bead Panel according to the manufacturer's instructions.

Cytokine signaling networks were evaluated using Ingenuity® Pathway Analysis (IPA®) (QIAGEN Bioinformatics.). Gene identifiers were substituted for each cytokine and mapped to their corresponding objects in the Ingenuity Knowledge Base. Networks were generated based on the ratio of median concentration of each cytokine in BMSA vaccinated group to adjuvant control group and their connectivity.

### Statistical analysis

All statistical analyses were performed using IBM SPSS (version 17, SPSS Statistics/IBM Corp., Chicago, IL, USA). For two-group comparison, *t*-test was used and the mean values of all variables were compared. For multigroup comparisons, one-way anova was used and followed by a *post hoc* test (Fisher protected least significant difference (PLSD) test) for two-group comparisons. *p* < 0.05 indicated a significant difference.

## Results

### Characteristics of BMSA

**Figure 1:**
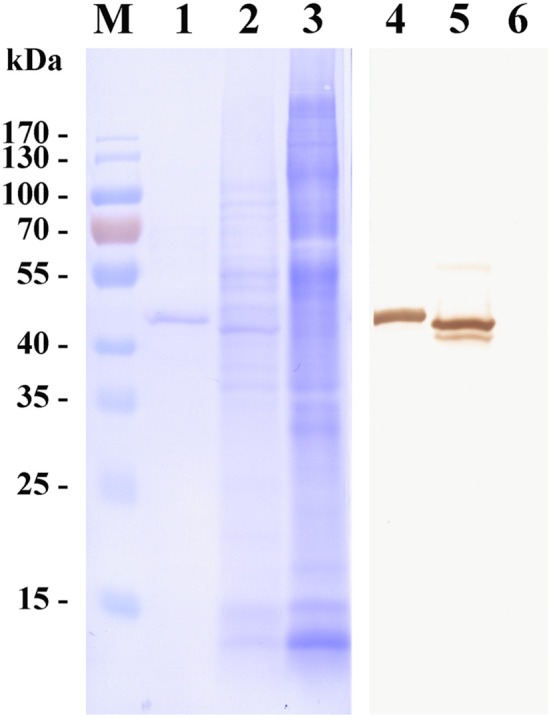
Western blot of a surface antigen of *Babesia microti* merozoites (BMSA). Recombinant BMSA with a His-tag (lane 1 and 4), *B. microti* crude proteins (lane 2 and 5), and normal red blood cell (RBC) proteins (lane 3 and 6) were analyzed. Protein was stained with CBB R-250 (lane 1–3); Protein incubated with an anti-BMSA monoclonal antibody. The molecular mass was 43 kDa (lane 4–6).

To investigate the characteristics of a merozoite surface antigen involved in the invasion of erythrocytes, a recombinant protein was produced. Polymerase chain reaction (PCR) amplification of cDNA from the mRNA of erythrocytes infected with *B. microti* yielded a 981 bp amplicon. The cDNA was predicted to encode a protein of 326 amino acid residues (pI = 5.21), consistent with the observed molecular mass of 43 kDa of the His-tagged recombinant protein (Figure 1). Fifty-three percent of the residues were negatively charged and 43% were positively charged. PredGPI (a GPI-anchor predictor)^1^ analysis predicted a carboxy-terminal sequence consistent with a glycosylphosphatidylinositol (GPI) anchor at residue 302. The transmembrane region (amino acid residues 309–326) was consistent with TMPred predictions^2^.

**Figure 2:**
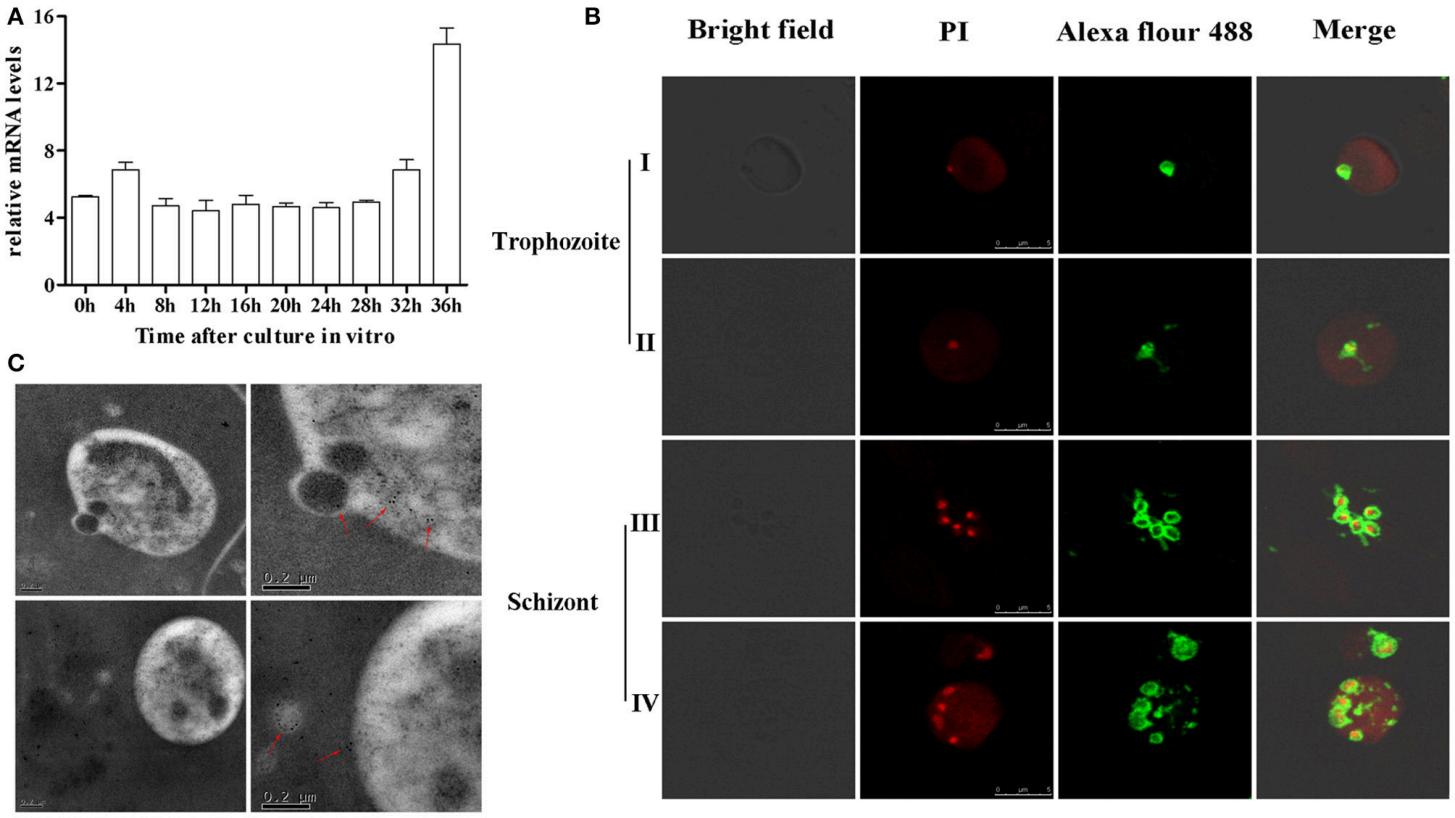
Expression and localization of BMSA in *Babesia microti*. **(A)** Transcriptional analysis of *bmsa* using quantitative PCR indicated that transcription of BMSA began early after parasite invasion and increased from 32 h to 36 h (schizonts) after synchronous culture. Results are presented as the mean ±*SD* of three independent experiments. **(B)** Immunofluorescence and laser confocal scanning microscopy indicated that BMSA was expressed during different phases. BMSA localized to the membrane of merozoites and to the cytoplasm of infected erythrocytes. Erythrocytes emit red fluorescence. **(C)** Immunoelectron microscopy images of BMSA showing that gold particles were located on the membrane of *B. microti* merozoites and in the cytoplasm of infected erythrocytes.

BMSA is a membrane-associated antigen of *B. microti*, although the exact subcellular location of BMSA is unknown. Transcription of *bmsa* during different phases, including ring forms, amoeboid forms, and merozoites, was detected using real-time PCR (Figure 2A). Transcription began soon after parasite invasion and increased in merozoites with a maximum transcription level occurring 36 h after synchronous culture. Expression of BMSA was observed in different phases using confocal laser scanning microscopy (Figure 2B) and immunoelectron microscopy (Figure 2C). Both techniques determined that BMSA was localized to the membrane of *B. microti* and revealed that BMSA was secreted into the cytoplasm of infected erythrocytes.

### Activity of serum antibodies induced by BMSA

**Figure 3:**
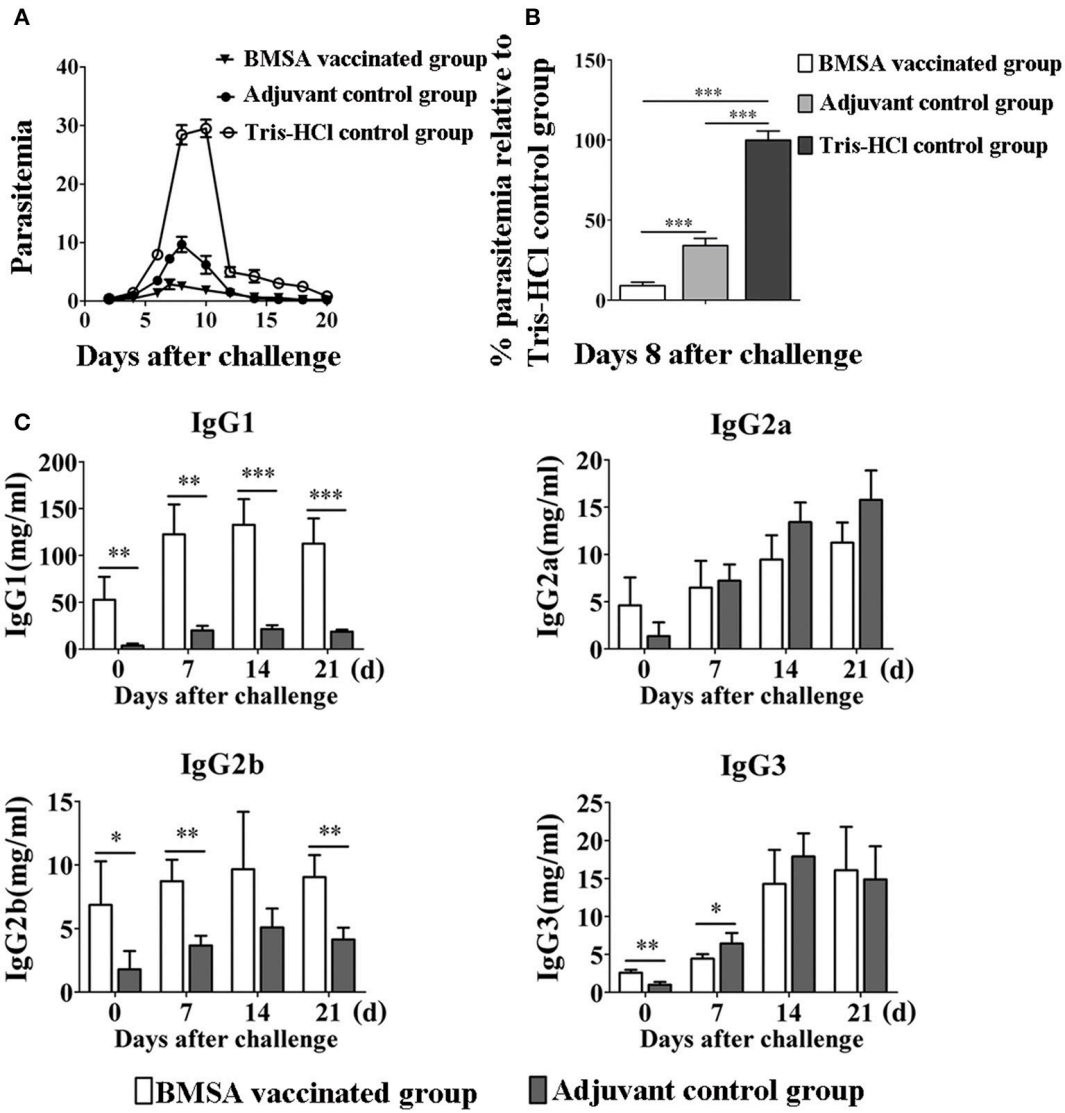
Changes in parasitemia and levels of immunoglobulin G of BALB/c mice vaccinated with BMSA. BMSA vaccinated or adjuvant control group BALB/c mice were challenged with 1 × 10^7^ erythrocytes infected with *B. microti* and were sampled on days 0, 7, 14, and 21. **(A)** Parasitemia was assessed every other day using flow cytometry (1,00,000 events). The data represent two individual experiments. **(B)** Parasitemia of the BMSA vaccinated group relative to the Tris-HCl control group on day 8 after challenge. **(C)** Levels of immunoglobulin G subclasses including IgG1, IgG2a, IgG2b, and IgG3 were detected using a Mouse Immunoglobulin Isotyping Magnetic Bead Panel (^*^p < 0.05; ^**^p < 0.01; ^***^ *p* < 0.001). The data represent two individual experiments.

To investigate the protective immune response induced by BMSA in the host, mice were vaccinated with BMSA and Freund's adjuvant (referred to here as BMSA vaccinated group), with Tris-HCl and Freund's adjuvant (referred to here as the adjuvant control group), or with Tris-HCl (referred to here as the Tris-HCl control group). After a challenge with 1 × 10^7^ infected red blood cells, the maximum level of parasitemia in the BMSA vaccinated group was approximately 5% and that in the adjuvant control group is 10% on day 8 after the challenge (Figure 3A). However, parasitemia in mice in the Tris-HCl control group reached a maximum of approximately 30% on day 8 after injection of *B. microti* (Figure 3A). The immune response induced by BMSA had a significant inhibitory effect that corresponded to 83.3% inhibition of invasion (*p* < 0.05) compared with that of the Tris-HCl control group (Figure 3B).

The serum level and subtype of antibody were detected using a Mouse Immunoglobulin Isotyping Magnetic Bead Panel. The serum levels of IgG1, IgG2b, and IgG3 were significantly elevated on day 0 after BMSA vaccinated compared with the levels in the adjuvant control group (Figure 3C). The level of IgG1 quickly and significantly increased until day 14 after the challenge in the BMSA vaccinated group. With challenge of *B. microti*, the levels of IgG2b gradually increased compared with those of the adjuvant control group. IgG2a and IgG3 both increased after the challenge but did not differ significantly between the BMSA vaccinated group and adjuvant control group (Figure 3C).

The level of antibodies in the serum of the BMSA vaccinated group increased as parasitemia worsened, and there was less severe parasitemia in the BMSA vaccinated group compared with that of the adjuvant control group after challenge with *B. microti*. The maximum percentage of infected erythrocytes differed significantly between the BMSA vaccination (5%) and the adjuvant control (10%) groups, suggesting that antibodies induced by BMSA, including IgG1 and IgG2b, inhibited *B. microti* from invading erythrocytes and reduced parasitemia.

### Changes in the levels of serum cytokines induced by BMSA

**Figure 4:**
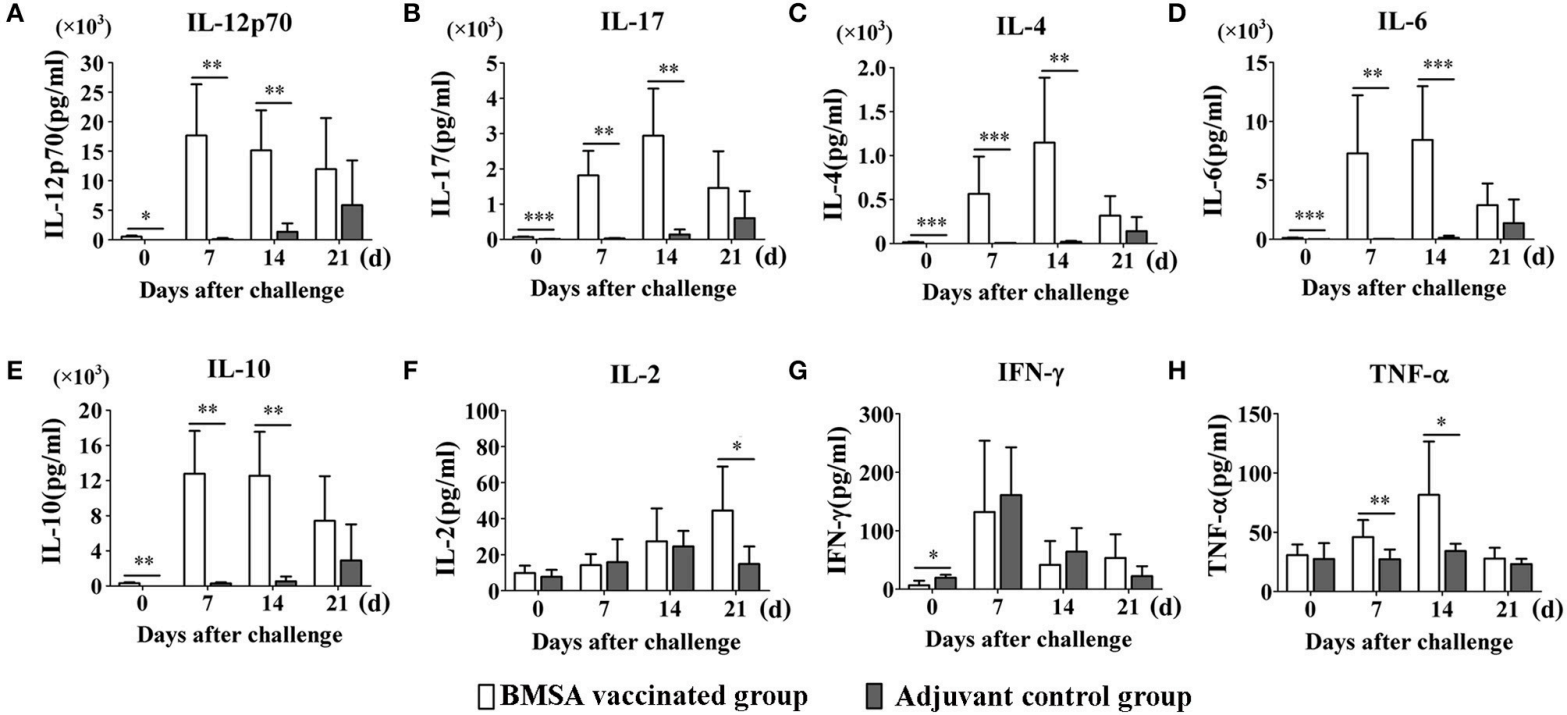
Cytokine levels in sera of mice immunized with BMSA or Tris-HCl. BALB/c mice of the BMSA vaccinated or adjuvant control groups were followed by a challenge with 1 × 10^7^ erythrocytes infected with *B. microti* and were sampled on days 0, 7, 14, and 21. **(A–H)** The levels of IFN-γ, TNF-α, IL-2, IL-12p70, IL-17, IL-4, IL-6, and IL-10 in serum were determined using a Mouse Cytokine/Chemokine Magnetic Bead Panel. The data represent two individual experiments (^*^p < 0.05; ^**^p < 0.01; ^***^p < 0.001).

To evaluate the protective immune response induced by purified recombinant BMSA against *B. microti* challenge, cytokines in the serum obtained from mice in the BMSA vaccination and adjuvant control groups were determined. After vaccination, the levels of IL-12p70, IL-17, IL-4, IL-6, and IL-10 in the BMSA vaccinated group were significantly higher compared with those in the adjuvant control group (Figures 4A–E). After challenge with *B. microti*, the levels of cytokines including IL-17, IL-4, IL-6, IL-10, IL-12p70, G-CSF, IFN-γ, TNF-α, IL-2, GM-CSF, MCP-1, MIP-1α, MIP-1β, and MIP-2 in the serum of the BMSA vaccinated group changed as infection progressed (Figures 4A–H; Supplementary Figures S1A–F). As parasitemia worsened, the level of the Th1 cytokine IL-12p70 in the serum of the BMSA vaccinated group quickly increased, peaked on day 7, and then decreased as parasitemia abated (Figure 4A). However, the levels of the Th1 cytokine TNF-α, the Th17 cytokine IL-17, the Th2 cytokines IL-4, IL-6, and IL-10, colony-stimulating factor G-CSF, and the chemokine MCP-1 all increased gradually, peaking 14 days after challenge (Figures 4B–E,H; Supplementary Figures S1A,C). These findings suggest that the serum levels of the Th1 cytokine IL-12p70, the Th2 cytokines such as IL-4, IL-6, and IL-10, and the Th17 cytokine IL-17 were critical for eliminating the parasite. The components of the protective immune response elicited by BMSA included Th1, Th2, and Th17 cytokines and other immune molecules that fight infection *in vivo*.

### *In Vitro* cytokine secretion by splenocytes stimulated with BMSA

**Figure 5:**
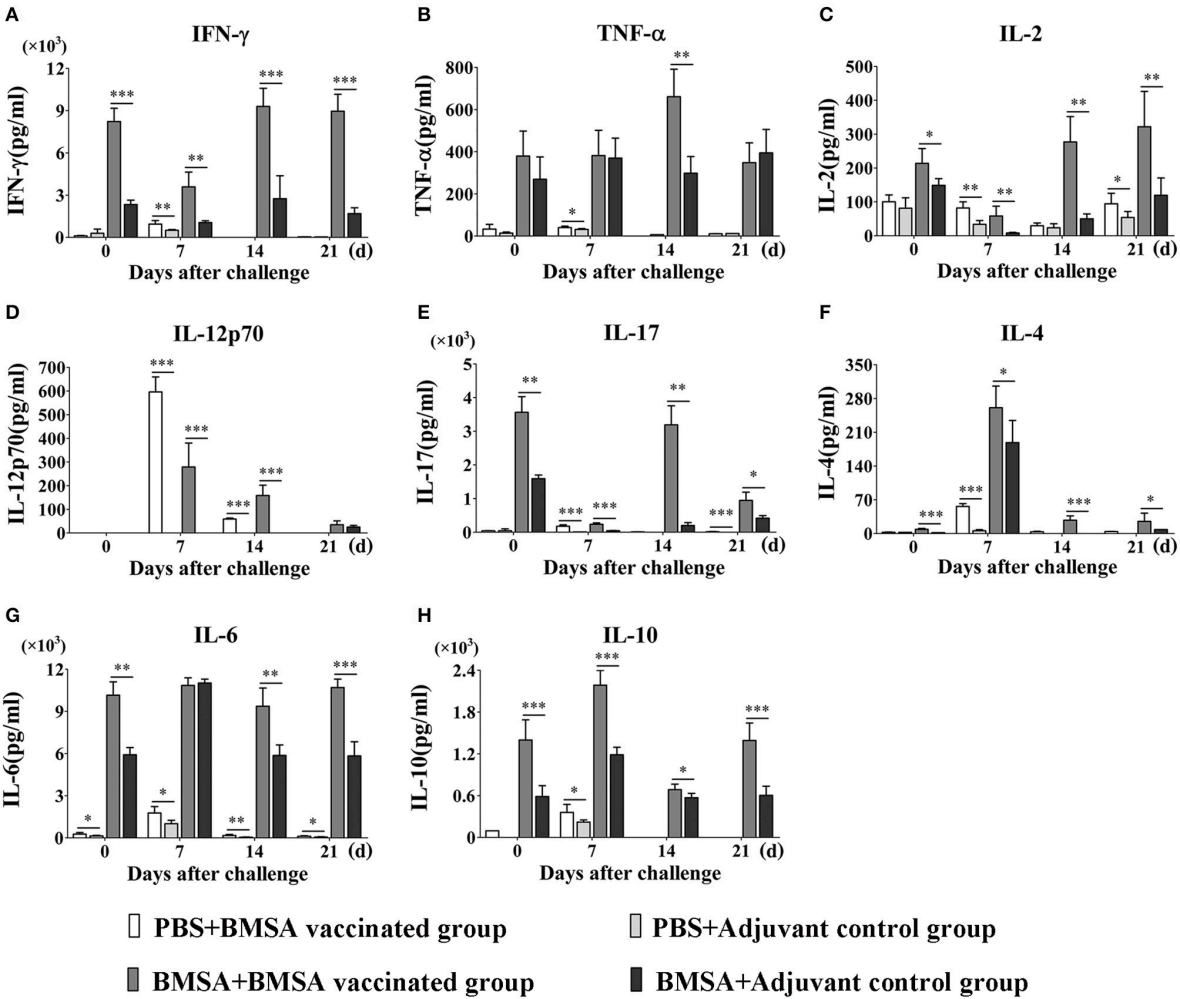
Cytokine levels in culture supernatants of splenocytes. BALB/c mice in the BMSA vaccinated or adjuvant control groups were followed by a challenge with 1 × 10^7^ erythrocytes infected with *B. microti* and were sampled on days 0, 7, 14, and 21. Splenocytes were cultured *in vitro* and stimulated with PBS, BMSA, or ConA (data not shown). **(A–H)** The levels of IFN-γ, TNF-α, IL-2, IL-12p70, IL-17, IL-4, IL-6, and IL-10 in culture supernatants were determined using a Mouse Cytokine/Chemokine Magnetic Bead Panel. The data represent two individual experiments (^*^p < 0.05; ^**^p < 0.01; ^***^p < 0.001).

To investigate the reason for the changes in the levels of serum cytokines, the expression of cytokines was examined in the culture supernatant of splenocytes stimulated with BMSA at different times. After vaccination with rBMSA, splenocytes were cultured and stimulated with rBMSA. The concentrations of Th1 cytokines such as IFN-γ and IL-2, Th2 cytokines such as IL-4, IL-6, and IL-10, and the Th17 cytokine IL-17 in the supernatant increased significantly in the BMSA vaccinated group compared with those in the adjuvant control group (Figures 5A,C,E–H). The concentrations of chemokines and colony-stimulating factors such as G-CSF, GM-CSF, MCP-1, MIP-1α, MIP-1β, and MIP-2 increased as well (Supplementary Figures S2A–F).

After challenge with *B. microti* and stimulation with BMSA, the concentrations of IL-12p70, IL-4, IL-6, IL-10, IL-2, IFN-γ, IL-17, MCP-1, MIP-2, G-CSF, GM-CSF, MIP-1α, and MIP-1β in the supernatant were significantly higher in the BMSA vaccinated group compared with those in the adjuvant control group (Figures 5A,C–H; Supplementary Figures S2A–F). As parasitemia worsened from days 0 to 7, the concentrations of IL-12p70, IL-10, and IL-4 in the supernatant increased in the BMSA vaccinated group (Figures 5D,F,H), although the concentrations of IL-2, IFN-γ, and IL-17 decreased (Figures 5A,C,E). The concentrations of chemokines and colony-stimulating factors such as MCP-1, MIP-2, and G-CSF gradually increased, peaking on day 7 (Supplementary Figures S2A,C,F). However, the concentrations of GM-CSF and MIP-1α remained unchanged (Supplementary Figures S2B,D).

Parasitemia abated between 8 to 21 days after challenge. During this time, the concentrations of IL-12p70, IL-4, and IL-10 in the supernatant decreased in the BMSA vaccinated group but the concentrations of IL-2, IFN-γ, TNF-α, and IL-17 slowly increased (Figures 5A–F,H). The concentrations of chemokines and colony-stimulating factors such as MCP-1, MIP-2, and G-CSF decreased, while the concentrations of GM-CSF and MIP-1α increased. After a challenge with *B. microti*, the elevated levels of IL-6 and MIP-1β in culture supernatants from the BMSA vaccinated group did not change (Figure 5G; Supplementary Figure S2E). This indicated that memory T cells were activated by the antigen and were secreting Th17 cytokines, Th2 cytokines (IL-4, IL-6, and IL-10), the chemokine MCP-1, and the colony-stimulating factor G-CSF. These substances accumulated and facilitated an immune response that reduced parasitemia.

### Relationship between Th17 and Th2 cytokines

**Figure 6:**
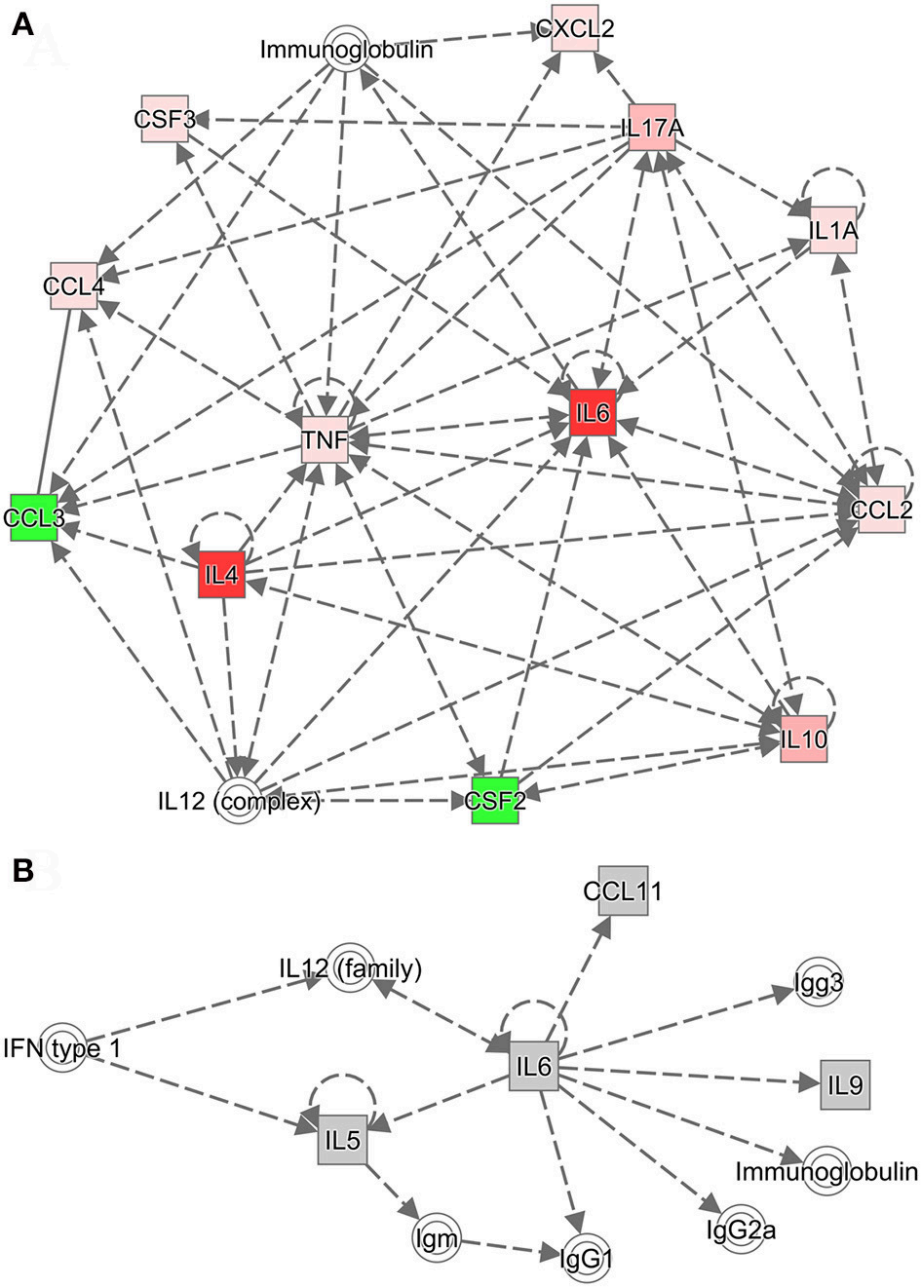
Cytokine networks were evaluated using Ingenuity Pathway Analysis (IPA). **(A)** The cytokine network according to IPA includes upregulated cytokines and their interactions. **(B)** The network connects the most upregulated cytokines and immunoglobulin G subclasses in the serum. Dotted lines indicate indirect associations.

Ingenuity Pathway Analysis was used to further investigate the relationships among cytokines and among chemokines and colony-stimulating factors. Cytokine networks contained the most upregulated cytokines and reflected considerable interaction in the serum after challenge with *B. microti*. Based on the results of the Ingenuity Pathway Analysis (Figure 6A), IL-2, IL-12, IL-17A (IL-17), and IL-4 regulated an increase in IL-10. However, IL-10 inhibited an increase in IFN-γ and increased IL-4, IL-6, IL-12, and IL-17. IL-2 increased IL-4, but IL-4 inhibited an increase in IFN-γ and increased IL-4 and IL-12. IL-2, IL-4, IL-6, IL-10, and IL-17A regulated an increase of IL-6. These findings indicate that increases in IL-4, IL-10, and IL-6 regulated humoral immunity. IL-6 promoted the production of serum IgG, including IgG1, IgG2a, and IgG3 (Figure 6B). IL-4 promoted an increase in serum IgG1, but inhibited the production of IgG2a and IgG3 (Supplementary Figure S3). After a challenge with *B. microti* and vaccination with rBMSA, memory T cells activated by BMSA secreted IL-17. This facilitated the secretion of IL-10, IL-4, and IL-6. The inflammatory response induced by infection increased IL-10 and IL-6, activating B cells to secrete IgG. Furthermore, IL-4 promoted a switching of the IgG subclass and increased the level of IgG1 in the serum. In summary, the protective immune response induced by BMSA was caused by memory T cells secreting IL-17, which mediated the Th2 immune response against infection.

### Therapeutic action of anti-BMSA monoclonal antibodies

**Figure 7:**
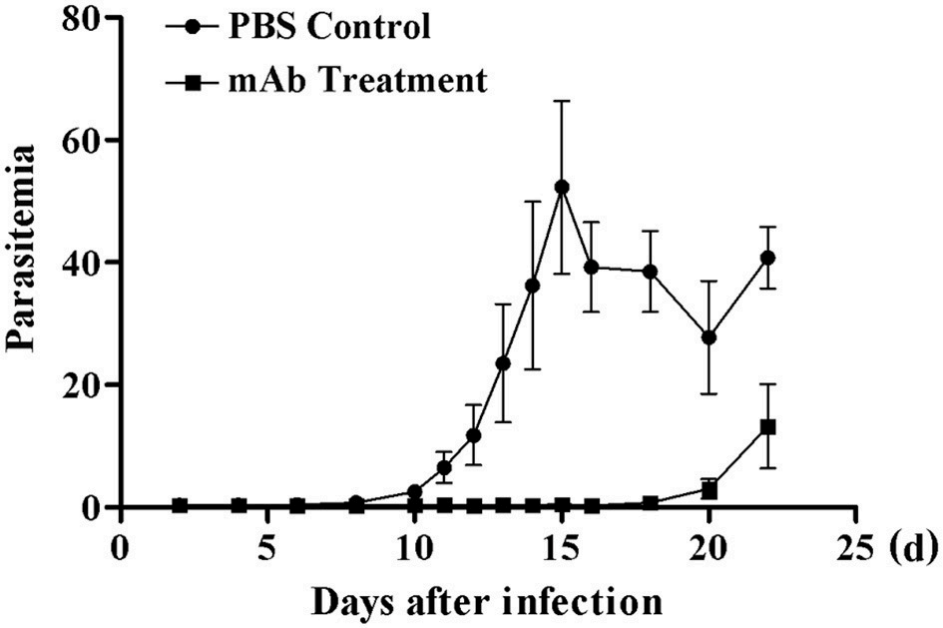
Parasitemia in NOD/SCID mice receiving anti-BMSA monoclonal antibody therapy. NOD/SCID mice were administered a total 5 mg of monoclonal antibody on days −1, 0, and +1 and were challenged with 1 × 10^5^ erythrocytes infected with *B. microti* on day 0. Parasitemia was measured every second day using flow cytometry after staining with SYBR Green I (1.00,000 events). The data represent two individual experiments.

The immune response induced by recombinant BMSA markedly inhibited the invasion of erythrocytes. We obtained nine monoclonal antibodies specific for BMSA that belong to IgG1, IgG2a, and IgG2b. To investigate the protection conferred by recombinant BMSA, one of the anti-BMSA monoclonal antibodies (IgG1) was transferred to NOD/SCID mice. In the phosphate-buffered saline (PBS) control group, the percentage of infected erythrocytes increased starting on day 8, peaked on day 15 with approximately 50% of erythrocytes being infected, and then decreased starting on day 16 (Figure 7). In mice treated with 5 mg of the monoclonal antibody, infected erythrocytes were detected until day 18 and subsequently increased, following a pattern similar to that of the control group (Figure 7). The monoclonal antibody had a half-life of 1–2 weeks and once the level of the antibodies in the serum decreased, the percent of infected erythrocytes increased. These results revealed that the presence of the anti-BMSA monoclonal antibody in the serum ameliorated parasitemia.

## Discussion

*B. microti* is a protozoan hemoparasite of the phylum Apicomplexa and is the causative agent of human babesiosis. There are diverse mechanisms through which the host defends against this parasitic attack, however, there have been very limited efforts made to understand the process of invasion of the erythrocytes by *B. microti* parasites and the many molecules associated with this process (Montero et al., 2009; Sun et al., 2011). The molecules that are involved in this process of invasion are likely to be important virulence factors (Alzan et al., 2016) that can be explored as potential candidates for vaccine development or to develop novel therapies, with the ultimate goal of preventing babesiosis and eradicating *Babesia* parasites. Our research presented here led to several interesting findings, some of which may have direct therapeutic application.

In the present study, a gene encoding the merozoite surface antigen was identified in *B. microti* parasites. Bioinformatic analyses showed that the BMSA sequences of *Babesia* parasites shared <30% identity and were absent in other apicomplexan parasites and metazoans. This indicates a conserved function for this protein in the *Babesia* species. It was confirmed that the expression of BMSA increased in mature merozoites and in the later stage of the life cycle. The protein is secreted into the cytoplasm of infected erythrocytes and transported toward the erythrocyte membrane. Using recombinant proteins, we show that BMSA was critical for parasite invasion of the host erythrocytes. In the first steps of erythrocyte invasion, *Babesia* species are similar to those of *Plasmodium* parasites that employ molecules located on the parasite surface coat to bind the host cell. In *Babesia bovis*, the merozoite surface antigen 2 plays an important role in the initial binding and invasion of host erythrocytes by the parasites (Mosqueda et al., 2002a). Consistent with this role, antibodies against such surface molecules would inhibit parasite invasion of the host erythrocyte. Through vaccine targeting, recombinant forms of the protozoa proteins have been successfully applied to protecting animal models against *Babesia* infection (Moitra et al., 2015; Gimenez et al., 2016b; Munkhjargal et al., 2016a). For instance, vaccination with BmAMA1 and BmRON2 simultaneously exhibited a limited protection against *B. microti* challenge and anti-BmAMA1 antibody inhibited the *in vitro* growth of *B. microti* parasites in RBCs by 80% (Moitra et al., 2015; Wang et al., 2017) The most important finding in the current study is the effective protection by vaccinated with recombinant BMSA, which can now serve as a vaccine to defend against the invasion of erythrocytes by *B. microti*. After a challenge with 1 × 10^7^ infected red blood cells, the maximum level of parasitemia in the BMSA vaccinated group was approximately 5% that corresponded to 83.3% inhibition of invasion compared with that of the Tris-HCl control group. Monoclonal antibodies specific to BMSA of *B. microti* parasites were evidenced by the measurement of antibody activation that inhibited the invasion of the erythrocytes in a NOD/SCID mouse model. These results indicate that rBMSA is a candidate antigen for a *Babesia* vaccine. This could be further developed to incorporate modifications that contribute to a multivalent vaccine, which could then be widely applied to babesiosis patients.

Similar to Plasmodium parasites, *in vivo* and *in vitro* evidence suggests that protective immunity to *Babesia* primarily involves cell-mediated responses. Immunization with BmMetAP1, BmHSP-70 or PROF elicited the modest protection from the infection of *B. microti*, owing to strong Th1 responses with high levels of IgG1 and Th1 cytokines (IFN-γ and IL-12) induced by these proteins (Terkawi et al., 2009; Munkhjargal et al., 2016a,b). However, humoral responses may also contribute to the anti-*Babesia* defense. Studies in BMSA vaccination animal models have established that *Babesia*-specific cytokine production is critically involved in the mechanisms of clearance of infection and host protection. It was known that the invasion of erythrocytes by *Plasmodium* parasites is a multistep process involving an interaction between receptors on the surface of erythrocytes (Reed et al., 2009; Fonseca et al., 2016) and MSP1, which is a component of a major complex on the merozoite surface of *P. falciparum*. This interaction induces cytophilic antibodies as well as CD4+ and CD8+ T cell responses that protect mouse models from malaria parasites. Therefore, once parasite infection is activated, B and T cells may participate in an immune response against *Plasmodium* as follows: (1) antibodies targeting the parasite toxins protect against disease; (2) T cells are important immunity effectors against the blood stage of malaria infections; and (3) cytokines released by CD4+ T cells are critical for the proliferation and maturation of B cells (Rénia and Goh, 2016). We hypothesized that after *Babesia* invades erythrocytes, B and T cells of the host may yield a protective immune response, which is similar to pathways of resistance to *Plasmodium* parasites. Recently, one study has described the protection elicited by MSA-derived vaccines from *B. bovis* and has shown that the anti-*Babesia* activity is due to CD4+ and CD8+ T cells (Gimenez et al., 2016b; Jaramillo Ortiz et al., 2016). In the present study, this protozoa's BMSA has been identified as an effector molecule that resulted in Th2 cytokines being secreted and contributing to BMSA-mediated protection in immunized BALB/c mice.

Moreover, IL-17 is a strong proinflammatory cytokine that acts on a wide-bound of immunocytes to induce the expression of cytokines, chemokines, and metalloproteases and is key in activating and attracting neutrophils to sites of inflammation (Banerjee et al., 2016). In our present study, live parasites or rBMSA significantly stimulated IL-17 production from peripheral blood mononuclear cells and spleen cells of BALB/c mice. This may further promote the anti-*Babesia* activity of macrophages and improve the Th1 response. The Th17 lineage plays an critical role in the clearance of specific types of pathogens that require a massive inflammatory response (Amedei et al., 2014). Often times, these pathogens are not adequately dealt with by Th1 or Th2 immunity (Korn et al., 2009). It is important to consider that IL-17 is a classic example of a “double-edged sword” that is capable of inducing a protective immune response to pathogen infection, while also having the ability to contribute to autoimmune disorders (Curtis and Way, 2009; Korn et al., 2009). IL-17-expressing CD4+ and CD8+ T-lymphocytes play significant roles in the inflammatory response to *Toxoplasma gondii* and *Trypanosoma cruzi*, thus blocking parasite invasion or replication in humans (Miyazaki et al., 2010; Silva et al., 2014). The importance of Th17 cells was demonstrated in their ability to control the development of such intracellular protozoa and promote the differentiation of activated macrophages and neutrophils, thereby supporting the inflammatory response. Our results show a correlation between partial and complete protection against infection with the local secretion of high levels of IL-4, IL-6, IL-10, and IL-17.

Classically, the Th2 cytokine responses are characterized by production of IL-4, IL-6, and IL-10. Th2 cells provide optimal help in generating humoral immune responses. In this study, IL-4, IL6, and IL-10 were mainly secreted by Th2 cells after immunized with BMSA. Given that B cells may require assist from T cells to accomplish their differentiation, IL-4 might effect on *Babesia* infections by inducing antibody generation by B cells when naïve B cells pass through the secondary lymph organs, most notably to the lymphonodus and spleen. The results indicate that secretion of IL-4 is an antigen-specific reaction induced by the vaccine and it might play a key role in protecting against *Babesia* infection in the mouse model employed here. In addition, IL-6 from Th2 cells and macrophages may influence adaptive immunity through the proliferation of and antibody secretion by B cells. Furthermore, we observed regulatory T cells or Th2 cell were induced to secrete IL-10. This affects the regulation and suppression of immune and inflammatory responses and indirectly inhibits Th1 cell development. Thus, *Babesia* antigen-specific T cells must induce antibody production, likely through cross regulation through cytokine mediated stimulation of B cells, resulting in a significantly higher serum level of IgG1, but not IgG2a, in BMSA vaccinated group than that in adjuvant control group. It is known that IgG1 is associated with Th-2 driven immunity while production of IgG2a is Th-1 related (Snapper and Paul, 1987; Finkelman et al., 1990). The Th2 cytokines induced by BMSA vaccination may have powerful effects on promoting the expression of IgG1.

The levels of a Th1 cytokine, IFN-γ, and IL-2 were increased in BMSA vaccinated group BALB/c mice and adjuvant control group BALB/c mice. There was no statistical difference in the levels of IFN-γ and IL-2 in the serum between BMSA vaccinated and adjuvant vaccinated mice. The cytokine profile and balance of cytokines were determined by characterization of the antigens. Mouse-associated cytokines in the culture supernatant of splenocytes derived from BMSA vaccinated were evaluated. The expression levels of IFN-γ and TNF-α in the culture supernatant of the splenocytes stimulated with BMSA increased more compared with those in the sera of BMSA vaccinated animal groups during all stages of infection. Significantly, upregulation of IL-6 was observed in the BMSA vaccinated animal groups 7 to 14 days after infection. A hypothesis was proposed to explain that BMSA has the ability to activate Th1 cytokines and pro-inflammatory cytokines. To validate this hypothesis, pathway analysis was performed to further investigate the inter-relationships between cytokine data sets in the Ingenuity Pathway database; we confirmed the hypohesis. The serum level of IL-6 from Tris-HCl control mice began increasing from day 12 and peaked on day 21 after challenge with *B. microti* parasites, indicating significant hysteresis. Similar results were observed for the expression levels of other cytokines in the Tris-HCl control mice when compared with adjuvant vaccinated mice. The results indicate that the expression levels of IFN-γ and IL-2, but not IL-6 and IL-4, were elevated in the Tris-HCl control or adjuvant vaccinated mouse splenocytes and had the potential for proliferation after infection of *Babesia* parasites or BMSA stimulation.

During the early stage of *Babesia* infection, IFN-γ concentrations increased in the sera of Tris-HCl control mice. IL-4 and IL-6 were undetectable, but high cytokine levels were detected ≥10 days after infection. These data may explain the upregulated expression levels of IFN-γ, TNF-α, and IL-6 in the BMSA vaccinated group. The IL-6 and IL-4 levels were low in the Tris-HCl control group, although they were high in BMSA vaccinated mice when splenocytes were activated by BMSA or serum from BMSA vaccinated mice. The data indicate that IL-6 and IL-4 may play a key role in protecting mice from *Babesia* infection. In animal models, 12 days after inoculation of the cavum abdominis with *Babesia*, macrophages began secreting cytokines that direct systemic inflammation and contribute to the pathophysiology of *Babesia* infection (Brown et al., 2015; Gimenez et al., 2016a,b). Furthermore, IL-6 and IL-4 play a pathogenic role in the persistence of *Babesia* infections through the inhibition of protective IFN-γ (Brown et al., 2015). After vaccination with BMSA, IL-4, IL-6, and IL-10 were mainly generated by Th2 cells. These cytokines may act to suppress the host macrophage activity, which upon the release of certain factors, are activated to act on invading pathogens and control excessive inflammation. IL-6 and IL-10 support the secretion of Th2 cells, which simultaneously activate B cells to induce systemic immune responses and produce antibodies to protect against *Babesia* infections. The activity of BMSA led us to further explore the possibility of using this molecule as an immunogen for vaccination. Using this approach, the parasitic load was reduced in the erythrocytes, and IL-6 and IL-10 levels were significantly upregulated. IL-10 is an anti-inflammatory factor that is critical for modulating inflammation in patients with severe malaria (Mahanta et al., 2015; Roussilhon et al., 2017). Thus, these results provide evidence that BMSA may induce a Th2 response, resulting in a cell-mediated immune response. *B. microti* antigen-specific T cells highly probably promote the production of anti-*Babesia*-specific antibodies, likely through the release of IL-6 and IL-10 and through contact-dependent stimulation of B cells. These data indicate that BMSA should be further explored as a potential candidate for developing babesiosis vaccines.

Freund's adjuvant activates both the innate and adaptive immune response, including the Th1 (CFA) and Th2 (IFA) response. Mycobacteria, which are intracellular bacteria responsible for the Th1 response attributed to CFA, also elicit Th17 cells in murine models *in vivo*. This is accompanied by elevated IL-6 levels (Brunner et al., 2010). Activation of numerous macrophages immunized with Freund's adjuvant plays a vital role in defending against *B. microti* infection (Li et al., 2012; Terkawi et al., 2015). This suggests that the decline of parasitemia in the adjuvant control group likely results from Freund's adjuvant.

In conclusion, we identified a BMSA protein that is present in *B. microti*. The protein is predominantly expressed in mature merozoites but may be expressed during other stages. The expression of this protein is associated with parasite invasion of the host erythrocytes and is required for the successful proliferation of the parasite in the host. Moreover, BMSA induced the resulting specificity of a cell-mediated immune response and a higher level of special IgG1 based humoral immunity. The levels of parasitemia significantly decreased after BMSA vaccination when mice were infected with babesia parasite (83.3% inhibition of invasion). In addition, monoclonal antibodies against this protein have provided effective protection against parasite infection, which supports the use of this *Babesia* protein as a potential candidate for developing a babesiosis vaccine; however, further studies are required and several are underway to further define the important steps toward developing an effective human babesiosis vaccine.

## Author contributions

All authors contributed to this work. XC: conceived and planned the research; SM and YF: performed the experiments; SM, YF, HG, MF, KQ, XL, and XC: analyzed the data; SM and XC: wrote the manuscript; YF and XC: edited the manuscript.

### Conflict of interest statement

The authors declare that the research was conducted in the absence of any commercial or financial relationships that could be construed as a potential conflict of interest.

## Footnotes

^1^http://gpcr.biocomp.unibo.it/predgpi/pred.htm

^2^http://www.ch.embnet.org/software/TMPRED_form.html

## Supplementary material

Supplementary Figure 1 Levels of chemokines in culture supernatants of splenocytes. BALB/c mice, in the vaccination or sham vaccination groups, followed by a challenge with 1 × 10^7^ erythrocytes infected with *B. microti*, were sampled on days 0, 7, 14, and 21. Splenocytes were cultured in vitro and stimulated with PBS, BMSA, or ConA (data not shown). **(A–F)** The levels of G-CSF, GM-CSF, MCP-1, MIP-1α, MIP-1β, and MIP-2 in culture supernatants were determined using a Mouse Cytokine/Chemokine Magnetic Bead Panel. The data represent two individual experiments. (^*^*p* < 0.05; ^**^*p* < 0.01; ^***^*p* < 0.001).

Supplementary Figure 2 Levels of cytokines in sera of mice immunized with BMSA or Tris-HCl. BALB/c mice, in the vaccination or sham vaccination groups, followed by a challenge with 1 × 10^7^ erythrocytes infected with *B. microti*, were sampled on days 0, 7, 14, and 21. **(A–F)** The levels of G-CSF, GM-CSF, MCP-1, MIP-1α, MIP-1β, and MIP-2 in sera were determined using a Mouse Cytokine/Chemokine Magnetic Bead Panel. The data represent two individual experiments. (^*^*p* < 0.05; ^**^*p* < 0.01; ^***^*p* < 0.001).

Supplementary Figure 3 The network of cytokines and immunoglobulins analyzed using IPA. The network connects the most up-regulated cytokines and immunoglobulin G subclasses in serum. Dotted lines indicate indirect associations.
