# Supplementary figures and images for: Evaluation of a Major Surface Antigen of Babesia microti Merozoites as a Vaccine Candidate against Babesia Infection
Source: Front Microbiol. 2017 Dec 19;8:2545. doi: 10.3389/fmicb.2017.02545 (PMC5742146; doi:10.3389/fmicb.2017.02545)

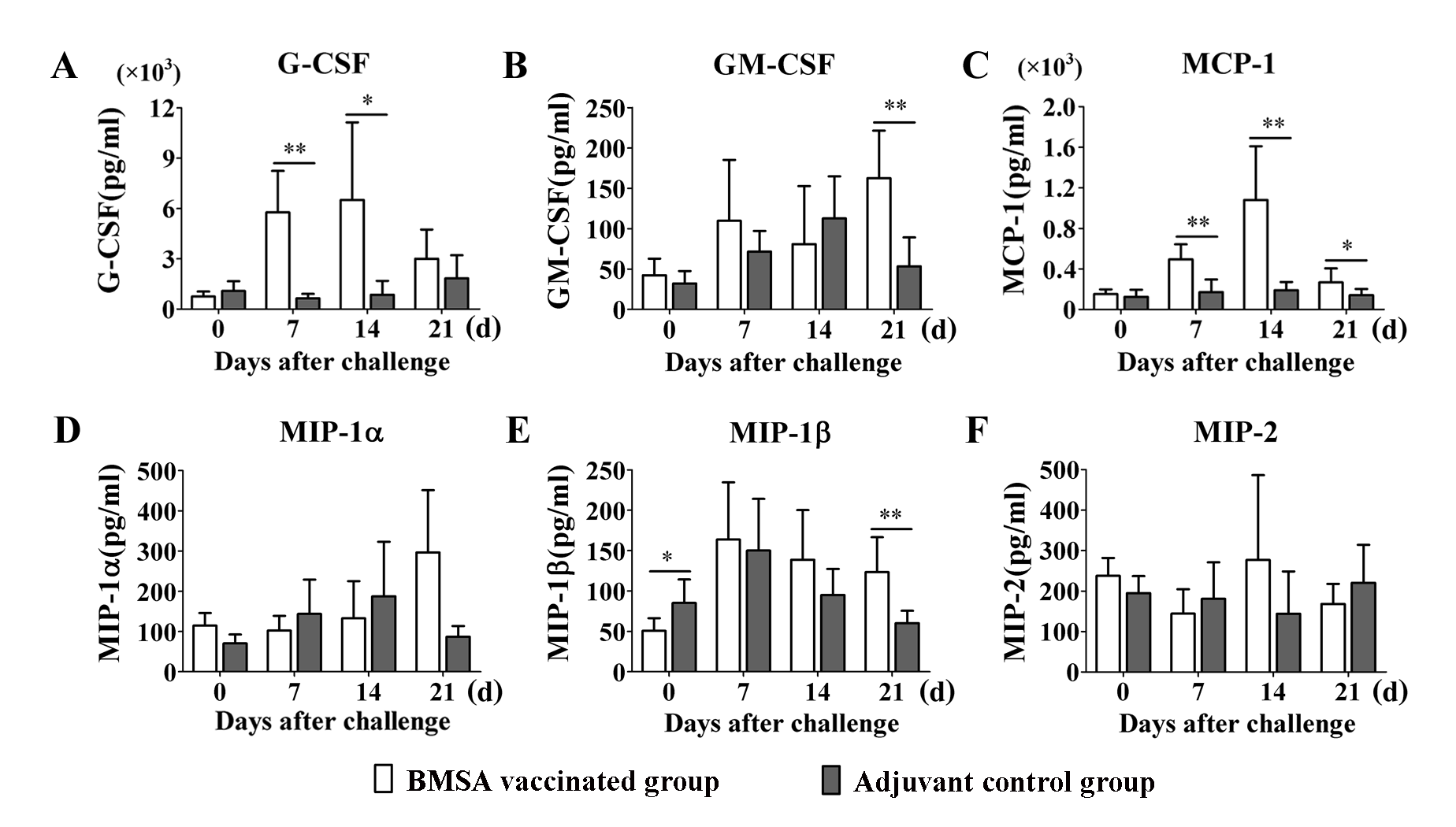

Supplement: Supplementary Figure 1 — Levels of chemokines in culture supernatants of splenocytes. BALB/c mice, in the vaccination or sham vaccination groups, followed by a challenge with 1 × 10^7^ erythrocytes infected with B. microti, were sampled on days 0, 7, 14, and 21. Splenocytes were cultured in vitro and stimulated with PBS, BMSA, or ConA (data not shown). (A–F) The levels of G-CSF, GM-CSF, MCP-1, MIP-1α, MIP-1β, and MIP-2 in culture supernatants were determined using a Mouse Cytokine/Chemokine Magnetic Bead Panel. The data represent two individual experiments. (*p < 0.05; **p < 0.01; ***p < 0.001). [file Image1.TIF]

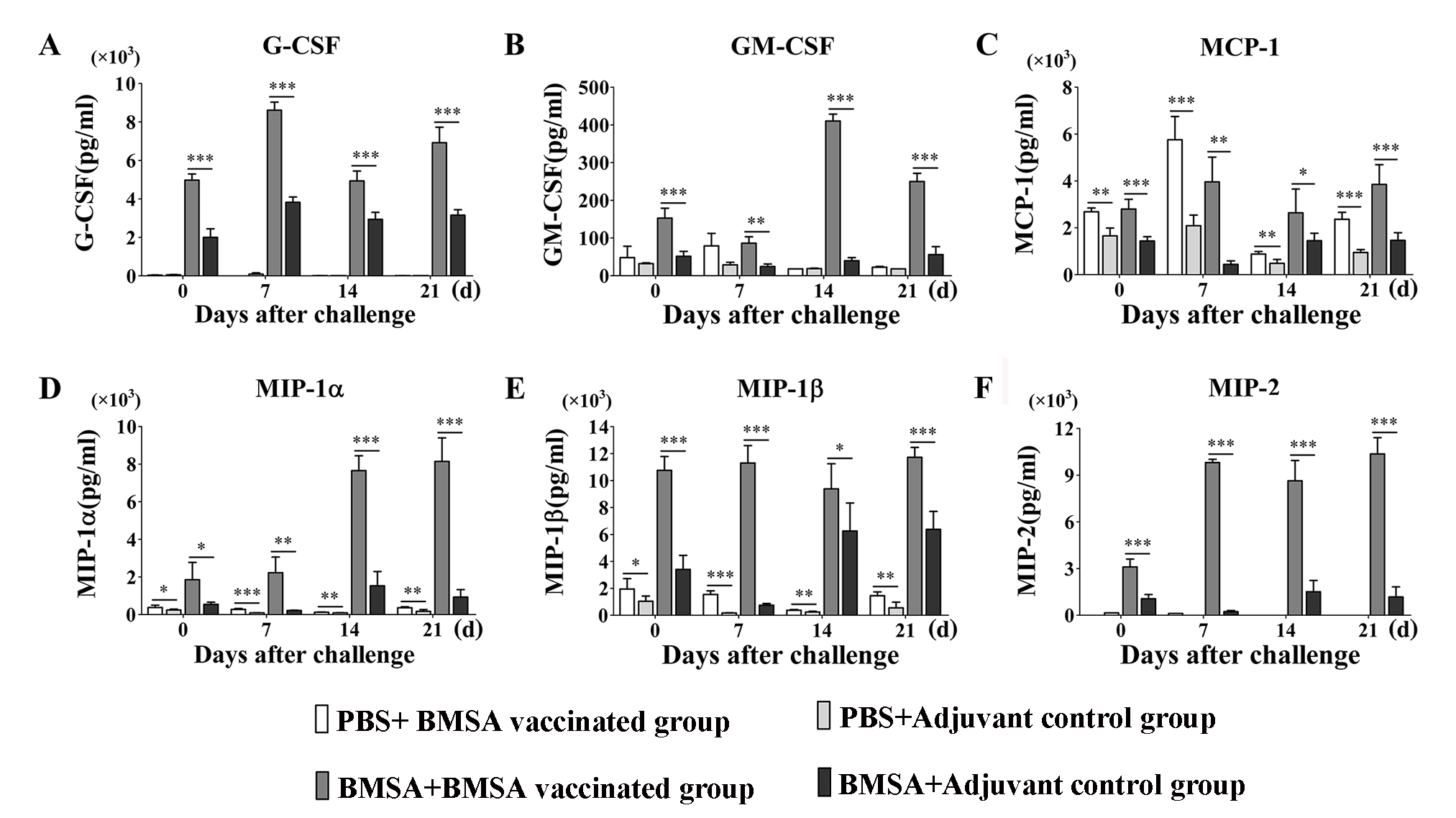

Supplement: Supplementary Figure 2 — Levels of cytokines in sera of mice immunized with BMSA or Tris-HCl. BALB/c mice, in the vaccination or sham vaccination groups, followed by a challenge with 1 × 10^7^ erythrocytes infected with B. microti, were sampled on days 0, 7, 14, and 21. (A–F) The levels of G-CSF, GM-CSF, MCP-1, MIP-1α, MIP-1β, and MIP-2 in sera were determined using a Mouse Cytokine/Chemokine Magnetic Bead Panel. The data represent two individual experiments. (*p < 0.05; **p < 0.01; ***p < 0.001). [file Image2.TIF]

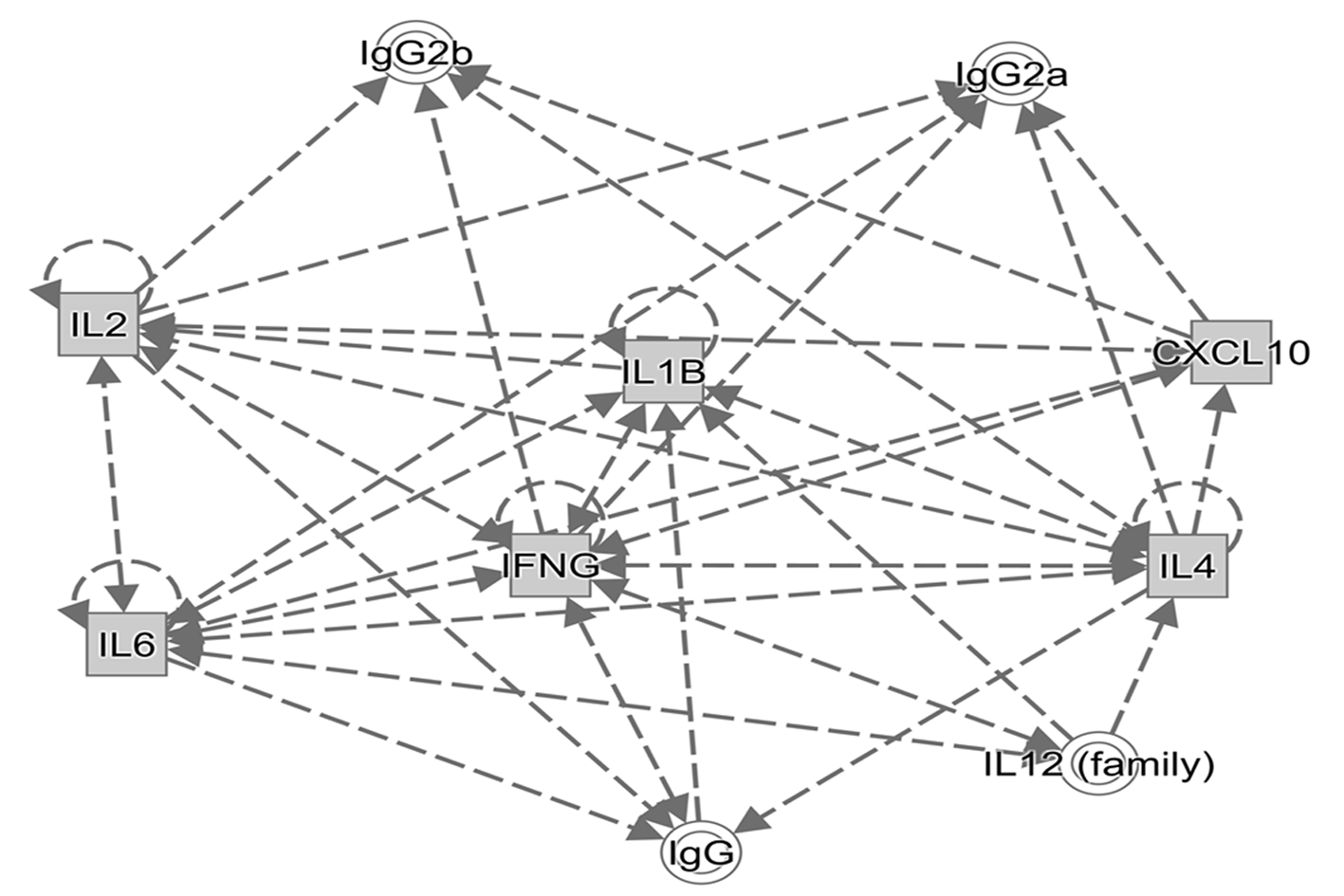

Supplement: Supplementary Figure 3 — The network of cytokines and immunoglobulins analyzed using IPA. The network connects the most up-regulated cytokines and immunoglobulin G subclasses in serum. Dotted lines indicate indirect associations. [file Image3.TIF]
